# Onychomycosis: experience of the laboratory of parasitology-mycology of CHU-Joseph Ravoahangy Andrianavalona, Antananarivo, Madagascar

**DOI:** 10.11604/pamj.2021.40.176.25216

**Published:** 2021-11-22

**Authors:** Norosoa Julie Zafindraibe, Fenosoa Anita Mireille Tsatoromila, Zolalaina Huberthine Rakotoarivelo, Njariharinjakamampionona Rakotozandrindrainy, Christian Rafalimanana, Olivat Aimée Rakoto-Alson, Lala Rasoamialy-Soa Razanakolona

**Affiliations:** 1 Paraclinic Unit of Formation and Research (UPFR), Parasitology - Mycology, CHU Joseph Ravoahangy Andrianavalona, Antananarivo, Madagascar,; 2 Faculty of Medicine, University of Antananarivo, Antananarivo, Madagascar,; 3 Regional Center for Blood Transfusion Analamanga, Antananarivo, Madagascar,; 4 National Reference Laboratory (NRL) for HIV/AIDS and Sexually Transmitted Infections, Antananarivo, Madagascar

**Keywords:** *Candida albicans*, *Trichophyton spp*, dermatophytes, onychomycosis, Madagascar

## Abstract

**Introduction:**

onychomycosis is defined as a fungal infection of the nails. They represent the main cause of onychopathy. They constitute a public health problem because of their increased prevalence in the world ranging between 20 to 30%. However, they remain under documented in Madagascar. This study was conducted in order to determine the mycological profile of onychomycosis diagnosed in the Parasitology Mycology laboratory of CHU-JRA.

**Methods:**

a descriptive retrospective study was taken over a 13-year period from June 2005 to December 2018. The data presenting onychomycosis on the mycological outcome register were included in the study. Results showing the presence of fungi on direct examination and / or culture were considered positive.

**Results:**

during the study period, a prevalence of 17.75% (180/1014) was observed. The age of our patients ranged from 3 to 76 years. Women were the most affected in 68.34% (n = 123) with a sex ratio of 0.46. Onychomycosis was localized in 64.65% of the fingers (n = 128). Simultaneous involvement of the hands and feet was found in 10% of the cases (n = 18). Candida albicans (C. albicans) was the most isolated in 33.03% (n = 71) of cases, followed by other species of Candida (24.65%, n = 53) and Trichophyton spp (9.3%, n=17). Mixed infections associating dermatophytes, Yeasts and molds were found in 23.33% (n = 42) of the cases.

**Conclusion:**

this is the first investigation dealing with onychomycosis in Madagascar. These data may be useful for future research and in the development of preventive and educational strategies.

## Introduction

Onychomycosis is a fungal nail infection caused by dermatophytes, non-dermatophyte moulds (NDMs), and yeasts or a combination there of [1]. In the last decades, the worldwide distribution of onychomycosis is on rise due to the ageing population, the climate changes, the changing lifestyle and the increasing number of immunosuppressed patients. Apart from the cosmetic problem, the disease has a significant negative impact on the physical, functional, psychosocial and emotional aspects of patients´ life [2]. Additionally, the infected nail serves as a chronic reservoir of infection, which can cause repeated mycotic infections of the skin and of the mucous membranes. Onychomycosis constitutes a frequent health problem with worldwide distribution. The incidence of the disease and the prevalence of the causative organisms vary between different parts of the world and change over time [3]. The global prevalence of onychomycosis is estimated to be around 5.5%, attributing to 50.0% of all nail disease cases [3, 4]. In Western countries, 80%-90% of onychomycosis cases are primarily caused by dermatophytes, with 5-17% due to yeasts and 2-3% due to NDMs (Non dermatophyte molds). In southern European countries, dermatophytes are causative organisms in 40-68% of cases, with 21-55% of cases due to yeasts. In Asian and Middle Eastern countries, dermatophytes account for 40-48% of cases, 43-46% due to yeasts, and 8-11% due to NDM-related infections. Comparatively, in Africa, onychomycosis-related infections are predominantly caused by yeasts [3]. In Madagascar, few studies have been carried out on these nail pathologies. The epidemiology of onychomycosis is yet to be fully established and no data are available on the incidence and common etiologic agents of onychomycosis. The aim of this work was to determine the mycological profile of onychomycoses diagnosed in the Parasitology - mycology laboratory of the Joseph Ravoahangy Andrianavalona University Hospital (CHU - JRA), Antananarivo, Madagascar.

## Methods

**Study design:** this was a descriptive retrospective study carried out at the paraclinical Training and Research Unit of Parasitology-Mycology of the CHU-JRA, in Antananarivo over a period of 13 years from June 2005 to December 2018. To carry out this study, the agreement of the head of department and the director of the JRA University Hospital were obtained. The biological and clinical data were kept strictly confidential.

**Participants:** over a period of 13 years (2005-2018), 1014 samples were obtained from patients with clinical suspicion of onychomycosis, who were referred to the Mycology department of the University Hospital of Antananarivo, Madagascar from community dermatologists or from the outpatient Dermatology Department of our hospital for mycologic examination in order to confirm the clinical diagnosis of onychomycosis.

**Inclusion and exclusion criteria:** all records of patients with onychomycosis as a result of the mycological examination were included in our study. The files selected had to mention the age, gender, requesting department, macroscopic aspect and location of the lesions and the results of the mycological examination. We collected files that contain as a reason for request a clinical suspicion of onychomycosis or as a result of onychomycosis. We took as a positive case any results of the mycological examination showing the presence of pathogenic fungi on direct examination and/or culture.

**Parameters of data analysis:** demographic characteristics (gender, age), predisposing factors and comorbidities (trauma of the nail, diabetes, psoriasis, immunosuppression, peripheral vascular diseases), location of onychomycosis (toenail/fingernail) and concurrent superficial fungal infection at other sites (such as tinea pedis, tinea cruris, tinea corporis, tinea manuum, tinea capitis and tinea faciei), were recorded for each patient.

**Statistical analysis:** the data were transcribed on the collection sheets and then entered into a Microsoft Office Excel file that includes all the parameters. The statistical analysis consisted of a description of our sample according to the characteristics mentioned above.

### Mycology investigation

#### Mycological sampling

Nail sampling for mycological examination was performed before any antifungal treatment, 15 days later for local antifungals and 1 month for oral antifungals. The sample was taken after cleaning the suspect nail(s) according to the recommendations of clinical practice. After cleaning the nails with alcohol 70% to remove contaminants. As sites of invasion and localization differ in different varieties of onychomycosis, separate approaches were taken to collect the nail specimens. The free edge of the nail was cut by a sterile nail clipper. Then, the powders under the nails were taken from the junction between healthy and pathogenic nail, scraped with a sterile scalpel blade. The resulting dander and fragments were then collected in wide-brimmed glass containers such as sterile petri dishes and sent to the laboratory for mycological analysis. In the case of paronychia, pus was also swabbed using a sterile swab. In leukonychia, a scratch is performed until the white area where the sample is taken is reached.

**Direct examination:** it consisted in deposing the samples taken on a slide, after thinning with 30% KOH and black chlorazole then covered with a slide. The preparation was read at objective 40 after 20 minutes of potash action. The entire preparations were microscopically examined by experienced personnel and were considered positive if the fungal elements (arthroconidia, blastoconidia, true hyphae or pseudohyphae) could be identified.

**Culture:** it was systematically performed on two types of culture media: Sabouraud dextrose agar with 5% of Chloramphenicol and Sabouraud dextrose agar with 5% of Chloramphenicol and Cycloheximide. One petri dish and one tube of each medium were seeded for each biological material collected and placed in the oven at +28°C if dermatophytosis was suspected and incubated at +37°C if candidiasis was suspected. The cultures were kept under observation for 4 weeks with regular monitoring (twice a week). The pathogenic role of moulds was only retained after several positive samples and only ifthey were found isolated on a culture medium.

**Identification:** the identification of the different species of filamentous fungi is based on the growth rate and especially on the macroscopic and microscopic aspects of the colonies. The identification of levuriform colonies was based on morphological and physiological characteristics (filamentation test or blast test). The filamentation test was performed by adding a drop of yeast suspension in 500 μL of fresh human serum. The preparation was incubated at +37°C for three hours and then spread between the slide and the lamella to observe under a microscope whether filamentation was present. The identification of dermatophyte species was carried out after an average of 30 days of incubation depending on the speed of growth, the macroscopic aspect on both sides of the colonies, the development and possible diffusion of pigment and their microscopic aspect.

## Results

### Population characteristics

**Figure 1:**
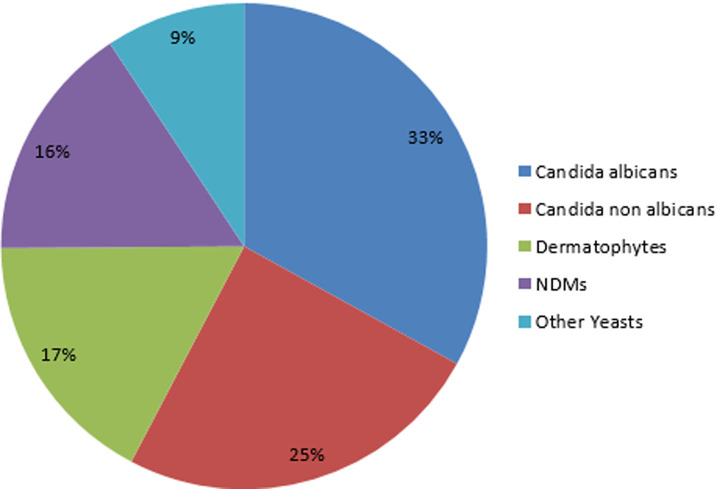
prevalence of different types of fungi in onychomycosis between 2005 - 2018

**Figure 2:**
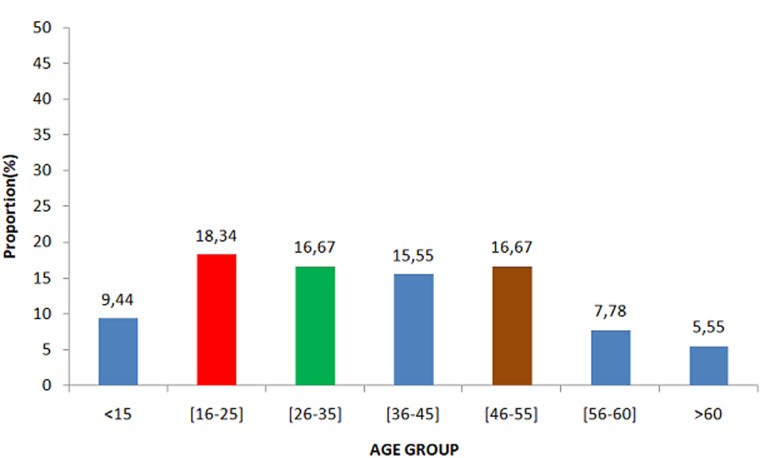
distribution of the study population by age group

**Figure 3:**
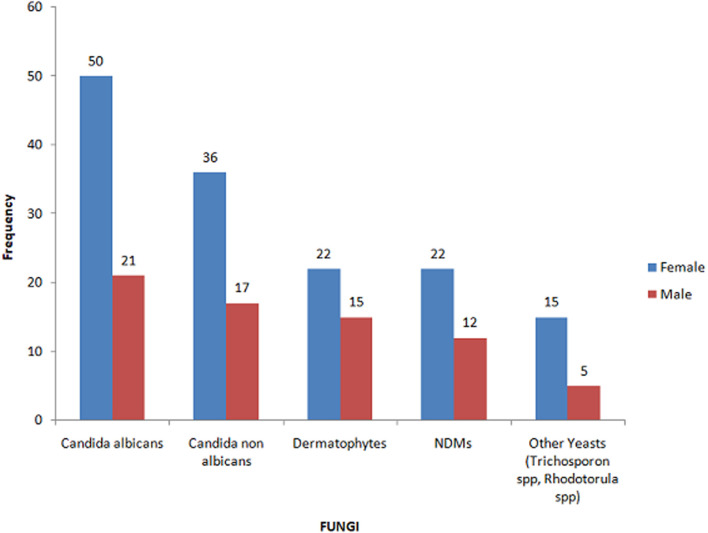
distribution of fungal species involved in onychomycosis by gender

This was a retrospective study, carried out over a thirteen years’ period, with patients referred to the parasitology and mycology laboratory for suspected onychomycosis. The study population consisted mainly of patients from the capital's university hospitals and dermatological clinics in Antananarivo. We counted 180 cases mentioning onychomycosis as a result, representing a frequency of 17.75% (180/1014) compared to other fungal infections during this period. Women were in the majority (123 cases or 68.34%) with a sex ratio of 0.46. *Candida albicans* is the most frequent species in 33.03% (n=72), followed by other Candida species in 24.65% (n=53) and dermatophytes in 17.20% of cases (Figure 1). The Figure 2 shows the distribution of fungal species involved in onychomycosis by gender. The average age was 36.29 years, with extremes of 3 and 76 years. Children less than 15 years of age made up 9.44% of the study population. More than half of the patients were adults between16 and 76 years of age (Figure 3).

**Distribution of onychomycosis by topography:** onychomycosis are located on finger nails in 64.65% (n=128) of patients and on toenails in 35.35% (n=70) (Table 1). Mixed finger-to-toe involvement was observed in 18 patients (10%).

**Table 1:** distribution of onychomycosis by type of infection

Type of Infections	Frequency	Proportion (%)
Mixed infection	42	23.33
One infection	138	76.76
	n=180	
Unilateral infection	162	90
Bilateral infection	18	10
	n=180	
Fingernails infection	128	64.65
Toenails infection	70	35.35
	n=198	

### Distribution according to mycological examination results

**Figure 4:**
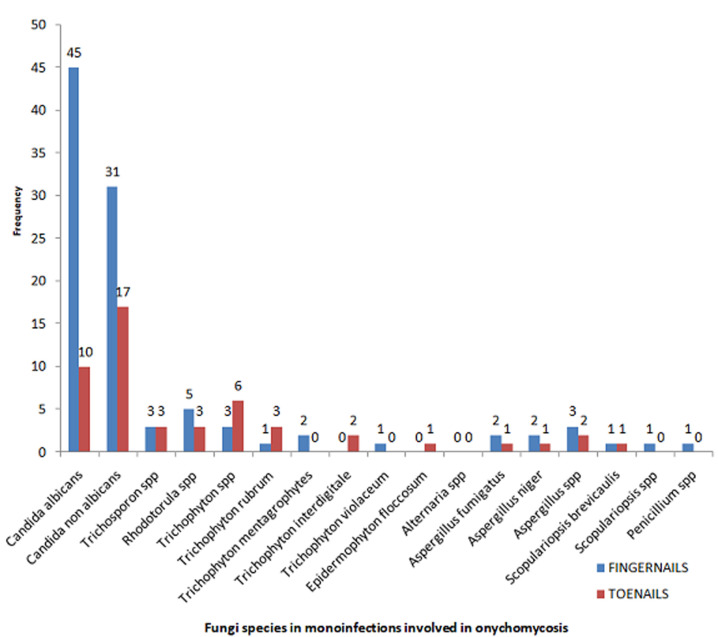
distribution of fungal species involved in monoinfections according to the site of onychomycosis

**Figure 5:**
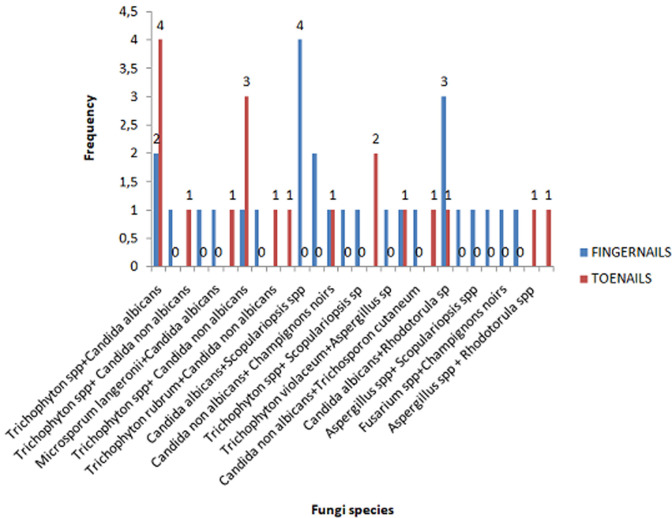
distribution of fungal species involved in mixed infections according to the site of onychomycosis

We encountered three groups of fungi whose yeasts were responsible for 66.98% of onychomycosis (*Candida albicans*, other species of Candida, other yeasts) (Figure 1). They were preferably located at the finger nail (Table 1, Figure 4). In mono infections, *Candida albicans* is the most common species found in 37.68 % of cases (n=52) followed by other Candida species in 31.17% (n=43). *Trichophyton spp* was observed in 6.52% of cases (n=9). Four cases of *Trichophyton rubrum*, two cases of onychomycosis with *Trichophyton mentagrophytes* and two cases with Trichophyton interdigitale were also observed (Table 2). Among mixed infections, an associated dermatophyte infection with yeast was observed in our study population. Among them, *Trichophyton spp* with *Candida albicans* in 9.53% (n=4) of cases, *Microsporum langeronii* with *Candida albicans* for one case (Table 2, Table 3). An association of dermatophyte infection with NDMs was also found in four cases. We also found onychomycosis caused by Non dermatophytes moulds such as *Scopulariopsis brevicaulis, Fusarium spp* or *Aspergillus spp*. Six species of NDMs were identified in onychomycosis (Figure 4, Figure 5). For hand onychomycosis, in mono infections, *Candida albicans* was the common etiological agents (n= 45), then the other species of in 31 cases, *Trichophyton spp* and *Trichophyton rubrum* was more observed in toenails (Figure 4).

**Table 2:** distribution of fungal species involved in monoinfections for onychomycosis

ONE INFECTION BY	Fungi species	Frequency	Proportion (%)
**YEASTS**	**Candida albicans**	52	37,68
	**Candida non albicans**	43	31,17
	**Trichosporonspp**	5	3,63
	**Rhodotorulaspp**	6	4,35
**DERMATOPHYTES**	**Trichophyton spp**	9	6,52
	**Trichophyton rubrum**	4	2,9
	**Trichophyton mentagrophytes**	2	1,44
	**Trichophyton interdigitale**	2	1,44
	**Trichophyton violaceum**	1	0,73
	**Epidermophytonfloccosum**	1	0,73
**NDMs**	**Alternariaspp**	1	0,73
	**Aspergillus fumigatus**	2	1,44
	**Aspergillus niger**	2	1,44
	**Aspergillus spp**	4	2,9
	**Scopulariopsisbrevicaulis**	2	1,44
	**Scopulariopsisspp**	1	0,73
	**Penicillium spp**	1	0,73
**Total**		138	100

* NDMs : Non Dermatophytes Moulds

**Table 3:** distribution of fungal species involved in mixed infections for onychomycosis

MIXED INFECTIONS	FUNGI SPECIES	Frequency	Proportion (%)
**YEASTS+DERMATOPHYTES**	**Trichophyton spp+Candida albicans**	4	9,53
	**Trichophyton rubrum+ Candida albicans**	1	2,38
	**Trichophyton spp+ Candida non albicans**	1	2,38
	**Trchophytontonsurans+Candida albicans**	1	2,38
	**Microsporumlangeronii+Candida albicans**	1	2,38
	**Trichophyton spp+Candida albicans**	1	2,38
	**Trichophyton spp+ Candida non albicans**	3	7,15
	**Trichophyton cutaneum+ Candida non albicans**	1	2,38
	**Trichophyton rubrum+Candida non albicans**	1	2,38
**YEASTS+NDMs**	**Candida albicans+ Aspergillus versicolor**	1	2,38
	**Candida albicans+Scopulariopsis spp**	4	9,53
	**Candida albicans+ Scopulariopsisbrevicaulis**	2	4,76
	**Candida non albicans+ Black moulds**	2	4,76
	**Candida non albicans+ Acremoniumsp**	1	2,38
	**Aspergillus spp + Rhodotorulaspp**	1	2,38
	**Black moulds +Rhodotorula spp**	1	2,38
	**Candida non albicans+Trichophyton sp+Scopulariopsi ssp**	1	2,38
**DERMATOPHYTES+NDMs**	**Trichophyton spp+ Scopulariopsi ssp**	1	2,38
	**Trichophyton verrucosum+ Black moulds**	2	4,76
	**Trichophyton violaceum+Aspergillus sp**	1	2,38
**YEASTS+YEASTS**	**Candida albicans+Trichosporon sp**	2	4,76
	**Candida non albicans+Trichosporoncutaneum**	1	2,38
	**Candida non albicans +Trichosporon sp**	1	2,38
	**Candida albicans+Rhodotorula sp**	3	7,15
**NDMS+NDMs**	**Aspergillus spp+ Scopulariopsis spp**	1	2,38
	**Aspergillus spp+Penicillium spp**	1	2,38
	**Fusariumspp+Black moulds**	1	2,38
	**Scopulariopsisspp+ Black moulds**	1	2,38
**TOTAL**		42	100

* NDMs: Non Dermatophytes Moulds

## Discussion

Onychomycosis is a chronic nail disease. Its prevalence in the general population varies from 2 to 18% [5], representing 18 to 50% of nail pathologies and constituting 1.5 to 18% of the reasons for consultation in dermatology [6]. This has a significant impact on the patient´s social, emotional and professional life. We found a frequency of 17.75% in our study. It is slightly higher than the prevalence reported in the literature, i.e. 6-9% in France [7], 2-18% in Latin America [8], and 9.2% in Tunis [9]. However, this prevalence seems to have increased in recent years. A prevalence of onychomycosis in young adults has been observed. More than half (50.28%) of the patients in our study were under 45 years of age. The average age was 36.29 years (Figure 2). This is consistent with the literature data, as found in Gabon [10] and Senegal [9] respectively 36.9 years and 34.2 years. The child under 15 years old was 9.4% in our study, in Tunisia 9.2% [9]. This corroborates the fact that onychomycosis is presented as an adult condition and is rare in children [11]. This low frequency in children could be explained by their different structure of their nail tablet compared to that of adults, their lower exposure to traumatism and their rapid nail growth [9, 12]. A female predominance (68.34%) was found in our study, with a sex ratio of 0.46. These results are comparable to those obtained in Gabon, Tunisia and France with 62.5%, 63.5% and 62.2% respectively [10-14]. This female predominance is explained by the more frequent contact with detergents during household activities for fingernails onychomycosis and by a recruitment bias. Indeed, the cosmetic concern is more expressed by women than men.

Concerning the topography of the lesions, our results do not allow us to confirm the hypothesis that onychomycoses are more frequent at the toenails reported by many authors [5, 14, 15]. Indeed, our study showed that 64.65% (Table 1) of cases were located on the fingernails, contrary to the surveys conducted in Senegal, Spain, Greece, and Pakistan [5, 15]. These results can be explained by patients´ negligence for this type of condition, which does not hurt. In general, yeast onychomycosis are less frequent than dermatophytes and are more common in warm climates [16] such as Madagascar. In our study, yeast onychomycosis and dermatophytes (Figure 1) were found in 66.98% and 17.20% of cases respectively. An Indian study also showed the same trend with 64.71% for yeast onychomycoses and 17.65% for dermatophytes [17]. In addition, yeast onychomycosis are more frequent than that of dermatophytes in finger nails and particularly affect women. *C. albicans* was the majority species (37.68%) in our study for mono infections (Table 2). This phenomenon can be explained, on the one hand, by the frequent humidity of women's hands due to household tasks and, on the other hand, byan aesthetic concern such as the care of repeated and aggressive manicures causing nail microtraumatisme. Paronychia was found to be the dominant clinical form in finger nails in the literature [5, 18].

The involvement of *Candida albicans* in onychomycosis varies from country to country. It can be responsible for 93% of Candida onychomycosis in Italy [19] or only 46.7%of cases in Argentina [20]. Its isolation in culture from fingernails is very suggestive of real candidiasis. Indeed, this yeast is not a component of healthy skin flora. On the other hand, the pathogenicity of other species of Candida (saprophytic flora of the skin) is uncertain in the nails. Only their presence in abundance and in successive samples is an argument for suspecting them to be responsible for onychopathy. However, since these yeasts can simply super infect underlying nail pathology, they are not necessarily responsible for the initial lesions. As for dermatophytes; they are preferably isolated at the feet.

In our study, the prevalence rate of onychomycosis caused by dermatophytes was 17.20%; the predominant species was *Trichophyton rubrum*, followed by *Trichophyton mentagrophytes* and *Trichophyton violaceum*. For onychomycosis caused by non-dermatophytes moulds, six species (*Alternaria spp, Aspergillus spp, Acremonium spp, Scopulariopsis spp, Fusarium spp, Black moulds*) have been found. This can be explained by the difficulty of asserting the pathogenicity of a mold basedon a set of arguments, including the renewal of the sample and their isolation in pure culture. Their pathogenic nature is retained only when direct examination is positive, when the culture is pure with growth at several seeding points and when it is repeatedly isolated on two separate samples.

In most cases, there is only one fungus in onychomycosis. Sometimes, however, two or more different pathogens share the nail tablet. In our study, 42 cases were found, including 15cases of association of a yeast with a dermatophyte; 4 cases of moulds with a dermatophyte; 4 cases of coinfection by NDMS, 12 cases of NDMs associated with yeast, and 7cases of coinfection by yeast (Table 3). Although the prevalence of onychomycosis was high in our study, not all only chopathy always means onychomycosis. The introduction of blind antifungal treatment may there forebe unnecessary, very costly and even responsible for undesirable side effects [21]. It is therefore recommended performing a mycological sampling of the suspect nail. The identification of the pathogenic fungus is important for the choice of antifungal agent. Our study has some limitations since it is a retrospective study, and the population studied is not representative of the entire Antananarivo population. Favorable factors, lesion characteristics and the patient's physical examination were not studied.

## Conclusion

Onychomycosis is a public health problem because of its high incidence and prevalence. Indeed, they are the main cause of onychopathy in Madagascar. Yeasts are the main cause of this condition in our study, especially in the fingers. *C. albicans* was the most isolated species in the fingers and the toes. Often unsightly, onychomycosis can affect a limb's functional prognosis through pain, recurrences or loco-regional infectious complications, and a risk of spreading the fungus to other parts of the body and other individuals is also important. Early and adequate management is therefore necessary, particularly in at-risk individuals. Detailed history, and clinical examination followed by mycological investigation are important for diagnosis, in order to select the optimal antimycotic agent targeting the responsible pathogen.

### What is known about this topic

- Onychomycosis is caused by various organisms, most often dermatophytes of the genus Trichophyton; other organisms include Candida, which is more common in fingernail infections;
- Onychomycosis affects toenails more often than fingernails because of their slower growth, reduced blood supply, and frequent confinement in dark, moist environments;
- In Madagascar, few studies have been carried out on the nail pathologies, no data are available on the incidence and common etiologic agents of onychomycosis.

### What this study adds

- In our study, we found a frequency of 17.75% of onychomycosis with a female predominance;
- Our study showed that 64.65% of cases were located on the fingernails; C. albicans was the majority species (33.03%);
- We found six species of non-dermatophytes moulds: Alternaria spp, Aspergillus spp, Acremonium spp, Scopulariopsis spp, Fusarium spp, Penicillium spp.
